# Notch1 promotes vasculogenic mimicry in hepatocellular carcinoma by inducing EMT signaling

**DOI:** 10.18632/oncotarget.12388

**Published:** 2016-10-01

**Authors:** Chen Jue, Cui Lin, Zhang Zhisheng, Qian Yayun, Jin Feng, Zhao Min, Wang Haibo, Shi Youyang, Tadashi Hisamitsu, Ishikawa Shintaro, Guo Shiyu, Liu Yanqing

**Affiliations:** ^1^ Institution of Combining Chinese Traditional and Western Medicine, Medical College, Yangzhou University, Yangzhou, Jiangsu, China; ^2^ Department of Oncology, the Second Peoples Hospital of Taizhou affiliated to Yangzhou University, Taizhou, Jiangsu, China; ^3^ Department of Physiology, School of Medicine, Showa University, Tokyo, Japan

**Keywords:** vasculogenic mimicry (VM), hepatocellular carcinoma (HCC), Notch1, epithelial-to-mesenchymal transition (EMT)

## Abstract

Hypervascularity is one of the main characteristics of hepatocellular carcinoma (HCC). However, the mechanisms of angiogenesis in HCC remain controversial. In this study, we investigate the role of Notch1 in angiogenesis of HCC. We found that Notch1 expression was correlated with formation of vasculogenic mimicry (VM) and expression of biomarkers of epithelial-to-mesenchymal transition (EMT) in the tumor specimens. Two HCC cell lines, HepG2 and MHCC97-H, with low and high Notch1 expression, respectively, were used to study the mechanism of VM formation both *in vitro* and *in vivo*. It was found that MHCC97-H cells, but not HepG2 cells form VM when they grow on matrigel *in vitro*. HepG2 cells gained the power of forming VM when they were overexpressed with Notch1, while knockdown Notch1 expression in MHCC97-H cells led to the loss of VM forming ability of the cells. Similar results were found in *in vivo* study. High expression of Notch1 in HepG2 promoted xenograft growth in nude mice, with abundant VM formation in the tumor samples. Moreover, we observed Notch1 was associated with the EMT and malignant behavior of hepatocellular carcinoma by analyzing clinical specimens, models for *in vitro* and *in vivo* experiments. HepG2 presented EMT phenomenon when induced by TGF-β1, accompanied by Notch1 activation while MHCC97-H with knockdown of Notch1 lost the responsiveness to TGF-β1 induction. Our results suggest that Notch1 promotes HCC progression through activating EMT pathway and forming VM. Our results will guide targeting Notch1 in new drug development.

## INTRODUCTION

Hepatocellular carcinoma (HCC) is the fifth most common malignant tumor with the third mortality worldwide [1]. It is a highly vascularized tumor required angiogenesis to grow, invade and metastasize [2]. Because of the advances in technology and clinical practice, survival yielded by resection has been improved in the last three decades. However, approximate 70% cases are detected with tumor recurrence in 5 years after resection and most of these cases are present in the first 2 years [3]. In patients who are not suitable for surgical resection, transarterial chemoembolisation and sorafenib are options as the systemic chemotherapy. However, marginal anti-cancer effect and non-benefit in survival have been proven by a number of clinical trials [3]. Therefore, elucidation of new mechanisms underlying the disease progression and identification of therapeutic targets could contribute to improve the disease control.

VM, proposed by Maniotis in 1999, is a type of vessel-like structures lined with high invasive tumor cells instead of endothelial cells [4]. In the last two decades, VM has been found to be present in various solid tumors and participating in nutrition and oxygen supply [5]. Some studies have suggested that VM is a risk factor for the poor prognosis in various malignant tumors such as prostate carcinoma, gastric carcinoma, glioma as well as pancreatic carcinoma [5]. EMT and stem-like differentiation are the key events underlying VM formation [6]. In the process of angiogenesis, various angiogenic modifying genes and embryonic genes are significantly increased or decreased [6]. Indentifying the change of these genes and understanding their mechanisms of actions will uncover new targets and provide anti-angiogenesis approaches in cancer therapy.

Notch is an embryonic gene and plays important role in cell fate decision during embryonic development [7]. In endothelial cell dependent vascularization, Notch signal cooperates with VEGF signal to construct a feed-back loop which plays important role in keeping the balance between vessel density and functions [8]. As an oncogene, Notch1 was found increased in many kinds of malignant tumors and associated with tumor cell proliferation, invasion and metastasis [9]. Recent studies demonstrated that Notch involved in the development of melanoma VM and potentially promoted VM formation via regulating NODAL signaling [10]. Results from another study indicated that Notch1 was upregulated in metastatic HCC and potentially involved in VM development in HCC [11]. However, the role of Notch1 in VM formation in HCC and related mechanisms was unclear. This study was conducted to explore the role of Notch1 in the development of VM in hepatocellular carcinoma and its underlying mechanisms.

## RESULTS

### Notch1 and its target gene Hes1 were over expressed in HCC tissues

**Figure 1:**
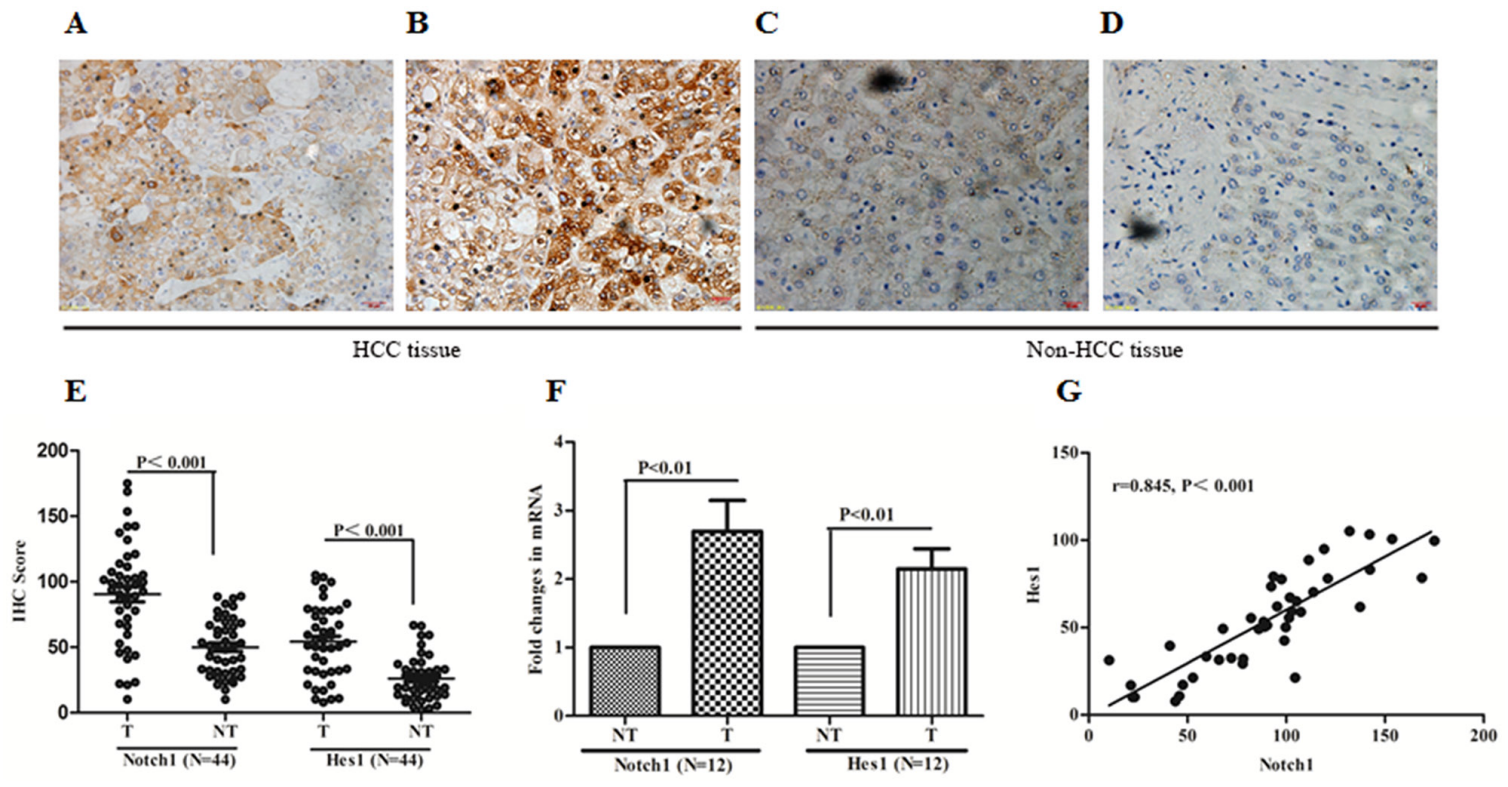
Notch1 and its downstream target Hes1 overexpressed in HCC specimens **A** and **B**. IHC of Notch1 and Hes1 in clinical specimens of HCC. **C** and **D**. IHC of Notch1 and Hes1 in adjacent non-tumor tissues. **E**. IHC score of Notch1 and Hes1 were compared between tumor and adjacent non-tumor tissues. **F**. Expression of Notch1 mRNA and Hes1 mRNA in 12 matched HCC and non-HCC tissues, expression was shown as fold changing. **G**. Notch1 expression was positively correlated with Hes1 expression in HCC tissues. All images were taken at magnification of 400×. Bar scale 20μm. T: tumor tissue, NT: non-tumor tissue.

To understand the expression of Notch1 and one of its downstream target gene, Hes1, in HCC tissues, the expression of Notch1 and Hes1 were investigated using immunohistochemistry (IHC) in 44 HCC specimens and matched adjacent non-tumor tissues. All tissue slides were digitally imaged and evaluated by Image Pro Plus (IPP, Media cybernetics, San Diego, CA), to avoid subjective bias. In the present study, all of the 44 tumor specimens were found to have moderate to strong positive Notch1 and Hes1 staining (Figure 1A and 1B), while 27 out of 44 matched paraneoplastic specimens were identified with only slight to moderate expression (Figure 1C and 1D). The staining of Notch1 and Hes1 were shown as a cytoplasmic pattern. As a target of Notch1, Hes1 expression was positively correlated with Notch1 in HCC tissues (r=0.845, P<0.001, Figure 1G). In HCC tissues, the median IHC score of Notch1 and Hes1 were 94.45 (10.25-175.08) and 49.77 (7.84-105.14), respectively. As a control, the IHC score in non-tumor tissues were 54.33 (10.15-88.93) and 21.76 (2.25-66.81) respectively. Significant difference of Notch1 and Hes1 were observed between tumor and non-tumor tissues (P<0.001, Figure 1E). To confirm the increasing tendencies of Notch1 and Hes1 in HCC tissues compared with adjacent non-cancer tissues, RT-PCR was performed in 12 matched tumor and non-tumor tissues to evaluate the expression in mRNA. As shown in Figure 1F, the results of RT-PCR were in accordance with IHC. These results suggested that Notch1 and its target gene Hes1 as oncogenes were overexpressed in HCC tissues.

### Expression of Notch1 and Hes1 were positively associated with VM in HCC tissues

**Figure 2:**
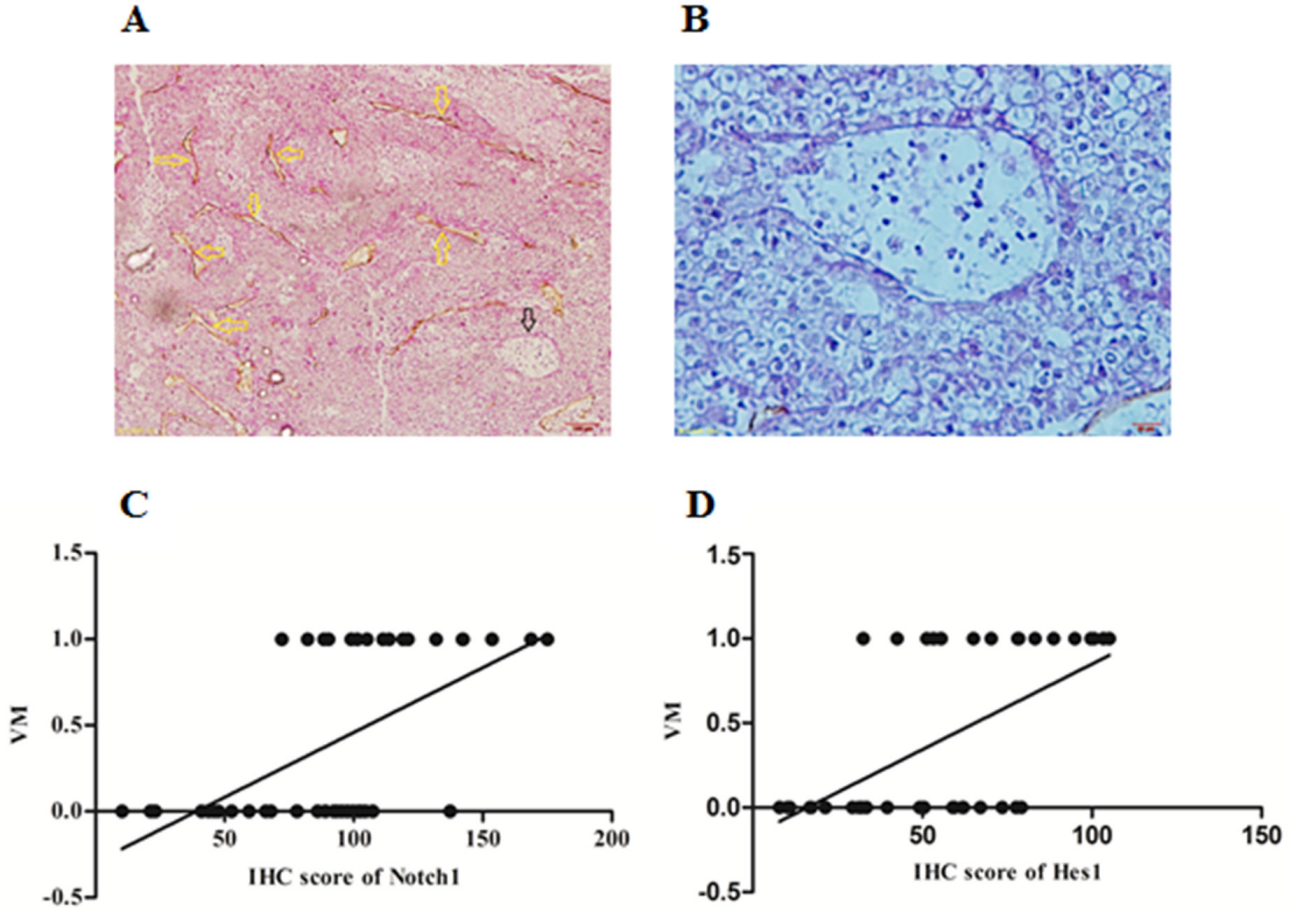
The expression of Notch1 and Hes1 was associated with VM formation in HCC tissues **A**. PAS-CD34 staining showed endogenous cells dependent vessels (EDV) and VM in one HCC specimen, yellow arrows indicated EDV, while black arrow indicated VM, original magnification was 200×. **B**. A large channel-like VM was detected in one HCC specimen, original magnification was 400×. **C** and **D**. Spearman correlation showed significant correlation between Notch1 and VM (r=0.590, *P*<0.001), Hes1 and VM (r=0.568, *P*<0.001). In C and D, numbers of Y-axis represented with and without VM, “0” represented without VM while “1” represented with VM.

In HCC tissues, basement membrane of blood vessels could be visualized using PAS histochemical staining while endothilal cells visualized by CD34 immunohistochemical staining. The PAS-CD34 double staining showed VM as a channel-like structure with negative CD34 and positive PAS staining (Figure 2A and 2B). Among total 44 samples, VM was detected in 17 (38.64%) cases. Spearman correlation analysis was further performed and the results indicated that Notch1 and Hes1 were positively correlated with VM (r=0.590, 0.568, P<0.001 and 0.001, respectively. Figure 2C and 2D). These findings indicated that VM formation or angiogenesis might be regulated by Notch1.

### Aberrant Notch1 expression and presence of VM indicated earlier post-operation recurrence

**Figure 3:**
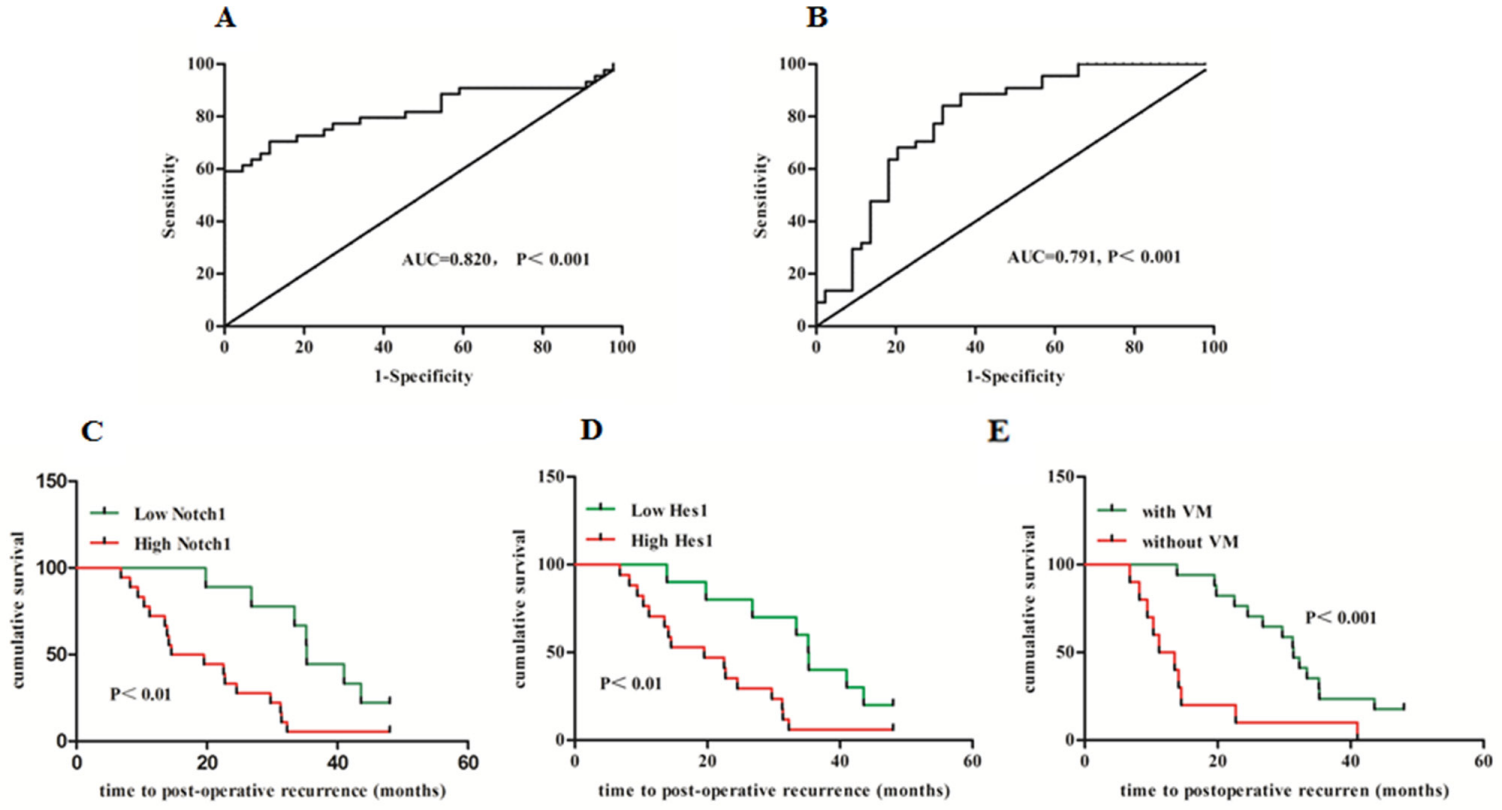
Aberrant Notch1 expression and presence of VM indicated earlier postoperative recurrence **A** and **B**: ROC statistics was employed to estimate the cut-points of the IHC score for Notch1 and Hes1 in tumor and non-tumor tissues. Kaplan-Meier curve and Log-Rank test were utilized to compare the time to postoperative recurrence in subgroups. **C**: High Notch1 VS. Low Notch1; **D**: High Hes1 VS. Low Hes1; E: With VM VS. Without VM.

To distinguish high and low expression of Notch1 and Hes1 in 44 HCC samples, ROC statistics was employed to estimate the cut-points of IHC score. As shown in Figure 3A and 3B, the IHC score of Notch1≥80.67 and of Hes1≥39.19 were considered high expression. In this study 27 of 44 cases were completed with 5-year post-resection following-up. As shown in Figure 3C and 3E, Kaplan-Meier analysis showed that high expression of Notch1 and Hes1, and exhibition of VM indicated earlier post-resection recurrence (median time to recurrence 20.12, 20.58 and 15.17 months, P<0.01, 0.05 and 0.01, respectively). These findings indicated that both increased Notch1 and presence of VM should be considered as risk factors for poor prognosis of HCC.

### Notch1 associated with invasiveness and VM potentiality of HCC cells

**Figure 4:**
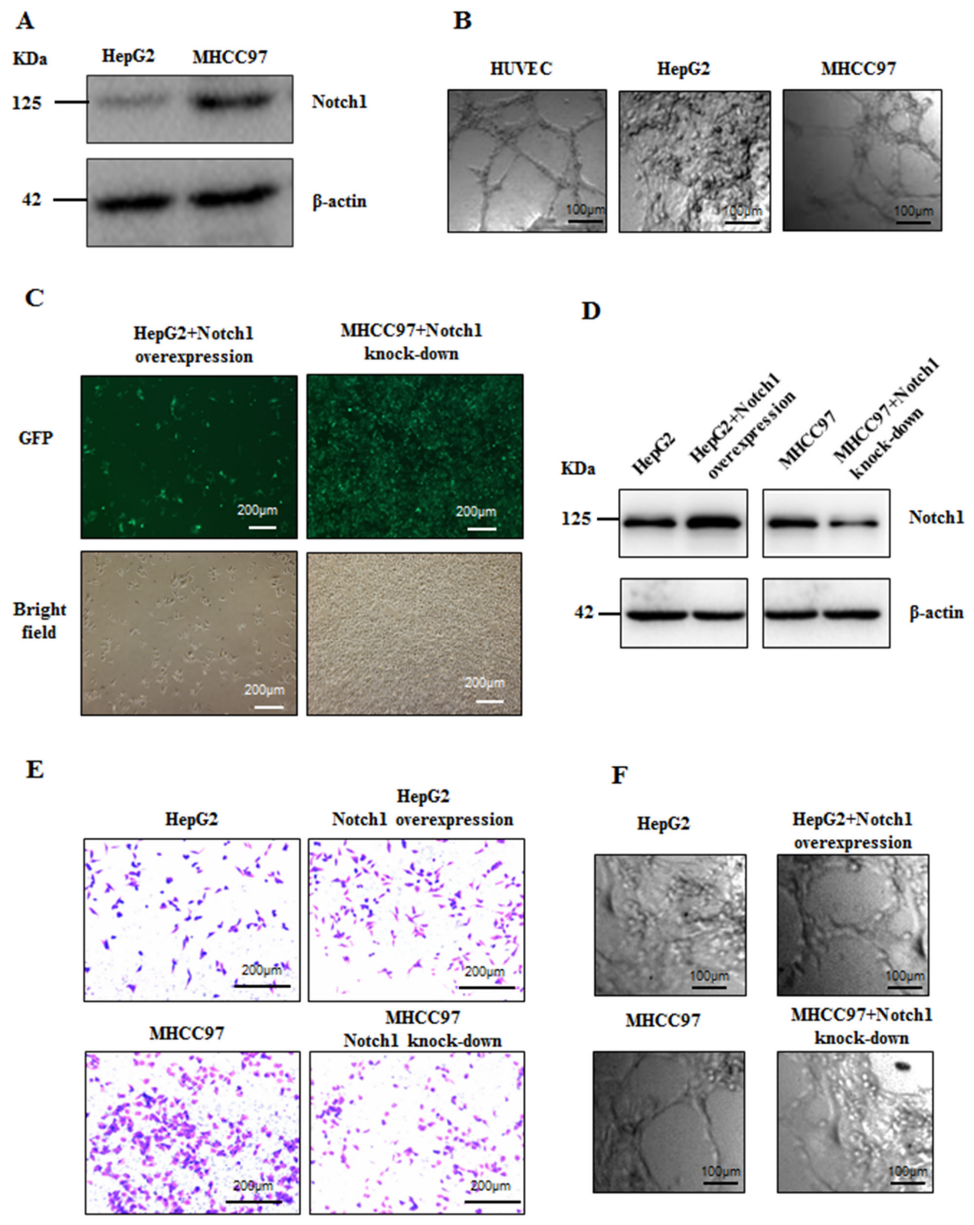
Notch1 associated with invasiveness and VM potentiality of HCC cells **A**. The expression of Notch1 is high in MHCC97-H and low in HepG2. **B**. MHCC97-H cells form VM, but HepG2 could not form VM when cultured in matrigel,. HUVEC was used as positive control. Original magnification was 100×. Overexpression for Notch1 in HepG2 and knock-down for Notch1 in MHCC97-H were mediated by lentivirus vector. **C**. Transduction efficiency confirmed by GFP. Original magnification was 100×; **D**. Stable overexpression or lowexpression of Notch1 in respective cell line were checked with Western Blot. **E**. Invasiveness of HepG2 was enhanced when overexpressed Notch1 while invasiveness of MHCC97-H was weakened when Notch1 was knockdown. Original magnification 200×. **F**. HepG2 formed VM when overexpressed Notch1 while MHCC97-H lost the VM forming ability when Notch1 was knockdown. Original magnification 100×.

It has been well known that VM normally formed in highly invasive tumors [5]. The formation of VM was investigated in two HCC cell lines, HepG2 and MHCC97-H, that represent low and high invasiveness respectively (Figure 4E). Firstly, we found that MHCC97-H cells with high Notch1 expression exhibit the ability to form tube-like structures while HepG2 cells with low Notch1 expression failed to form such structures (Figure 4A and 4B). To validate the role of Notch1 in VM formation, two lentiviruses were utilized to overexpress and knock-down Notch1 in HepG2 and MHCC97-H cells respectively. Successful overexpression and knock-down of Notch1 were confirmed by GFP signal and Western blotting (Figure 4C and 4D). Increased invasiveness and capacity of tube-formation was achieved by Notch1 overexpression in HepG2 cells, while knock-down of Notch1 in MHCC97-H cells destroyed their ability to invade and form tube-like structures (Figure 4E and 4F). These results suggested that Notch1 played important role in VM formation in HCC.

### Notch1 expression was elevated in TGF-β1 induced EMT model

**Figure 5:**
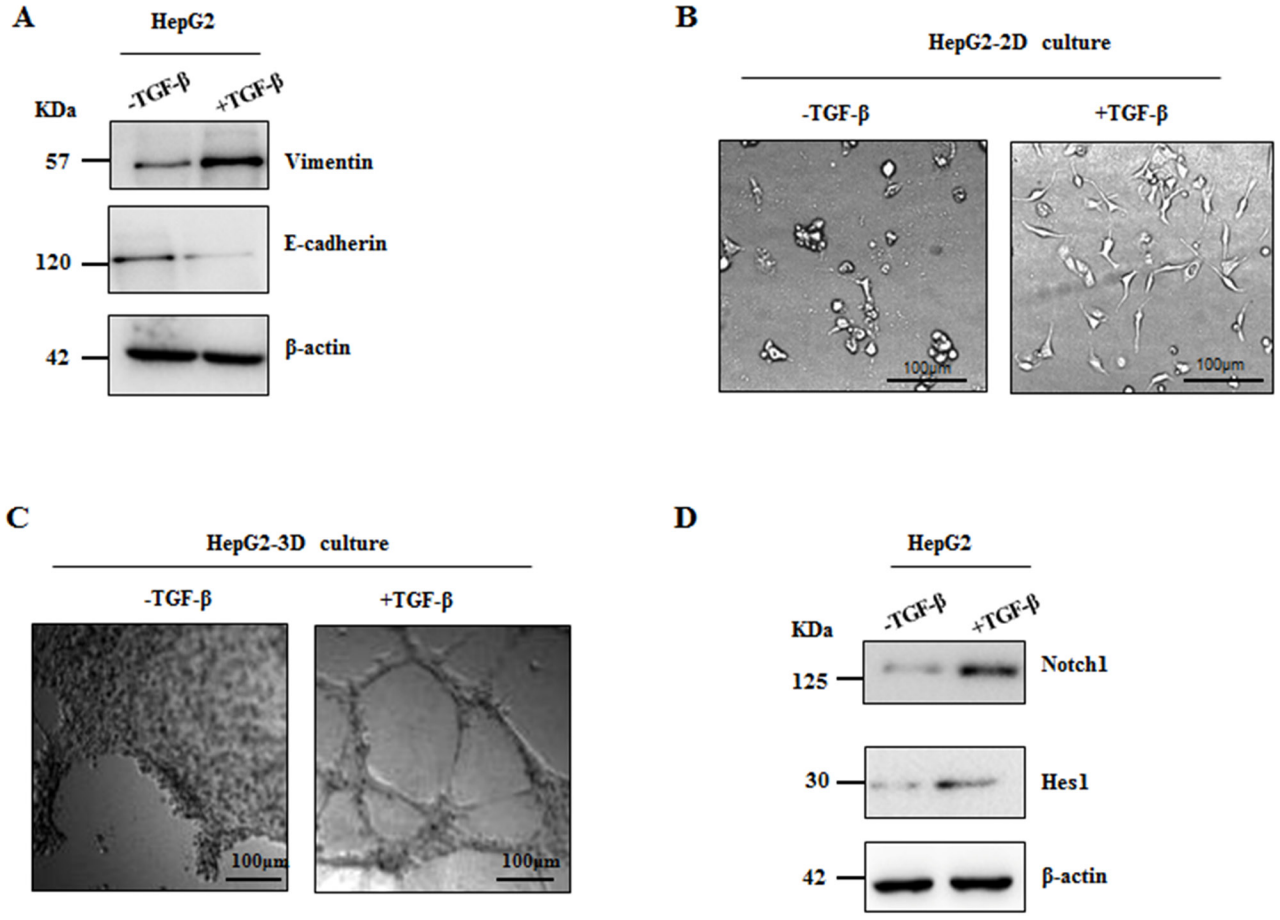
Notch1 expression was elevated in TGF-β1 induced EMT Treatment of TGF-β1 for 24 hours was used in HepG2 cells to induce EMT. **A**. Western blot showed increased Vimentin expression and decreased E-cadherin expression; **B**. Morphological change of HepG2 was observed, original magnification 200×. **C**. HepG2 cells formed VM induced by TGF-β1, original magnification 100×. **D**. stimulation of TGF-β1 increases expression of Notch1 and Hes1 in HepG2 cells.

It has been well documented that acquisition of mesenchymal phenotype of tumor cells from epithelial cells is the key step during VM formation [12]. As such, we speculated that Notch1 contributing to VM formation is associated with EMT. In this study, a classic *in vitro* EMT process was achieved by TGF-β1 treatment in HCC cells. After maintaining in 15ng/ml TGF-β1 for 24 hours, HepG2 cells revealed significant changes in the expression of EMT related biomarkers including the increase of vimentin and the decrease of E-cadherin (Figure 5A). Furthermore, morphological change of HepG2 cells such as elongation and spindle-like shapes were observed in 2-dimension cultural system (Figure 5B), while tube formation was found in 3-dimension condition (Figure 5C). Interestingly, we found that Notch1 and Hes1 were significantly increased in those TGF-β1 treated cells (Figure 5D), suggesting a possible role of Notch1 in EMT and consequent VM formation.

### Notch1 overexpression led to EMT in HCC cells

**Figure 6:**
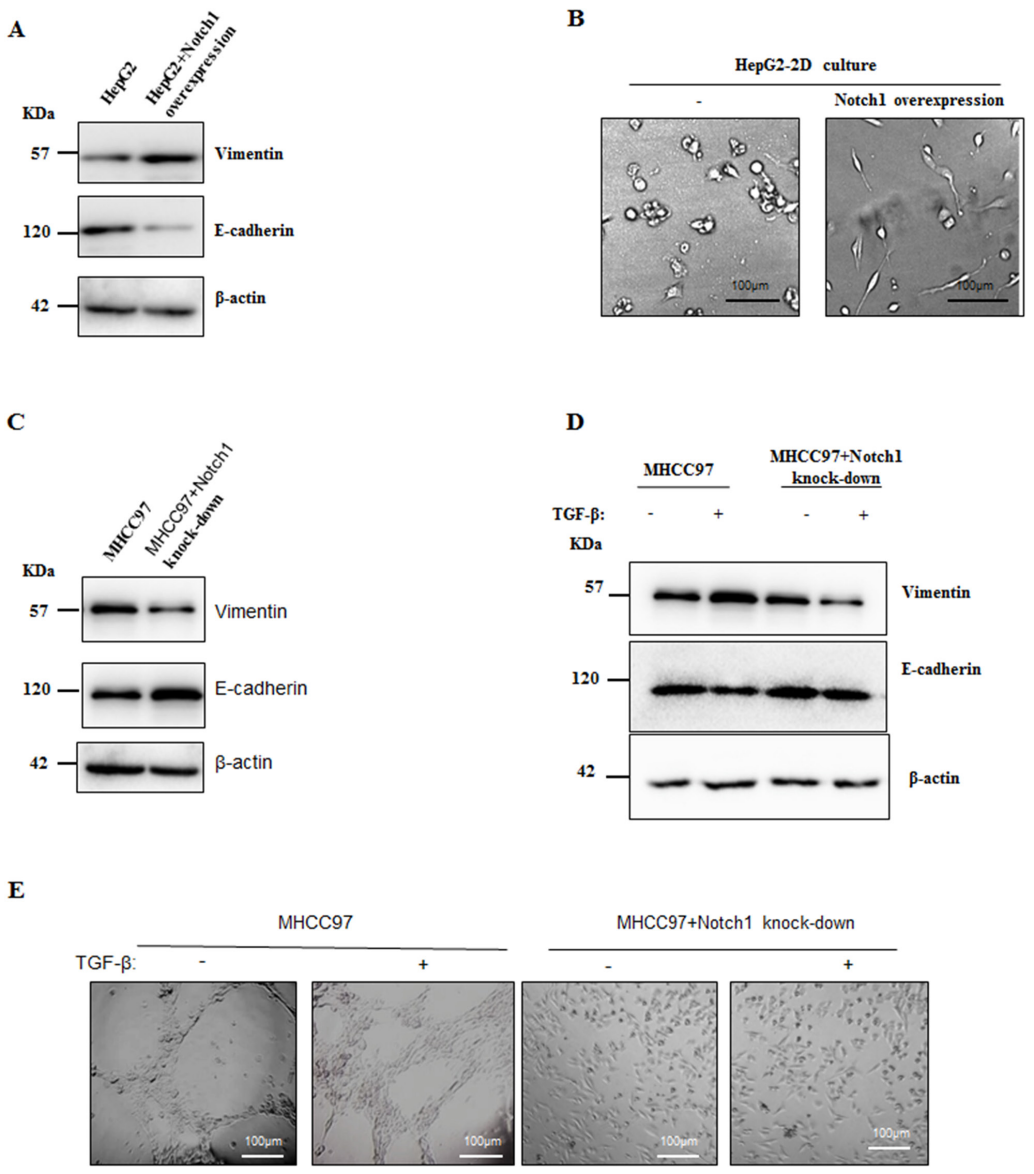
Notch1 overexpression led to EMT in HCC cells **A**. Overexpression of Notch1 induced HepG2 to obtain mesenchymal phenotype. Western Blot showed increased Vimentin and decreased E-cadherin. **B**. Morphological changes of HepG2 when Notch1 was overexpressed. Original magnification 200×. **C**. Knockdown of Notch1 induced increase E-cadherin and decrease Vimentin in MHCC97-H. **D**. Treatment of TGF-β1 for 24 hours induced increase E-cadherin and decrease Vimentin in MHCC97-H cells, while such effect was not observed when Notch1 was knockdown. **E**. TGF-β1 enhanced VM formation in MHCC97-H, but no VM formation was observed in Notch1 knockdown cells. Original magnification 100×.

Because Notch1 and Hes1 were increased in HepG2 after treatment with TGF-β1, we next determined whether overexpression of Notch1 promotes VM formation via inducing EMT. As shown in Figure 6A and 6B, overexpression of Notch1 in HepG2 cells induced the same morphological transition as the description above and led to the increase of vimentin expression and the decrease of E-cadherin expression. On the contrary, MHCC97-H cells with Notch1knock-down revealed significant decrease in vimentin and increase in E-cadherin (Figure 6C). We further assessed the TGF-β1 responsiveness in MHCC97-H cells and it's counterpart of Notch1 knock-down, including the expression of Vimentin and E-cadherin and VM formation ability. After stimulation with TGF-β1 for 24 hours, the ability of MHCC97-H to form VM was doubled, while it's counterpart failed to form VM (Figure 6E). Treatment with TGF-β1 for 24 hours increase Vimentin and decrease E-cadherin in MHCC97-H (Figure 6D). However, MHCC97-H with Notch1 knock-down lost the responsiveness to TGF-β1 induction (Figure 6D). Collectively, these results suggested that Notch1 induced-VM formation is mediated by the EMT process.

### Notch1 associated with EMT related biomarkers in HCC tissues

**Figure 7:**
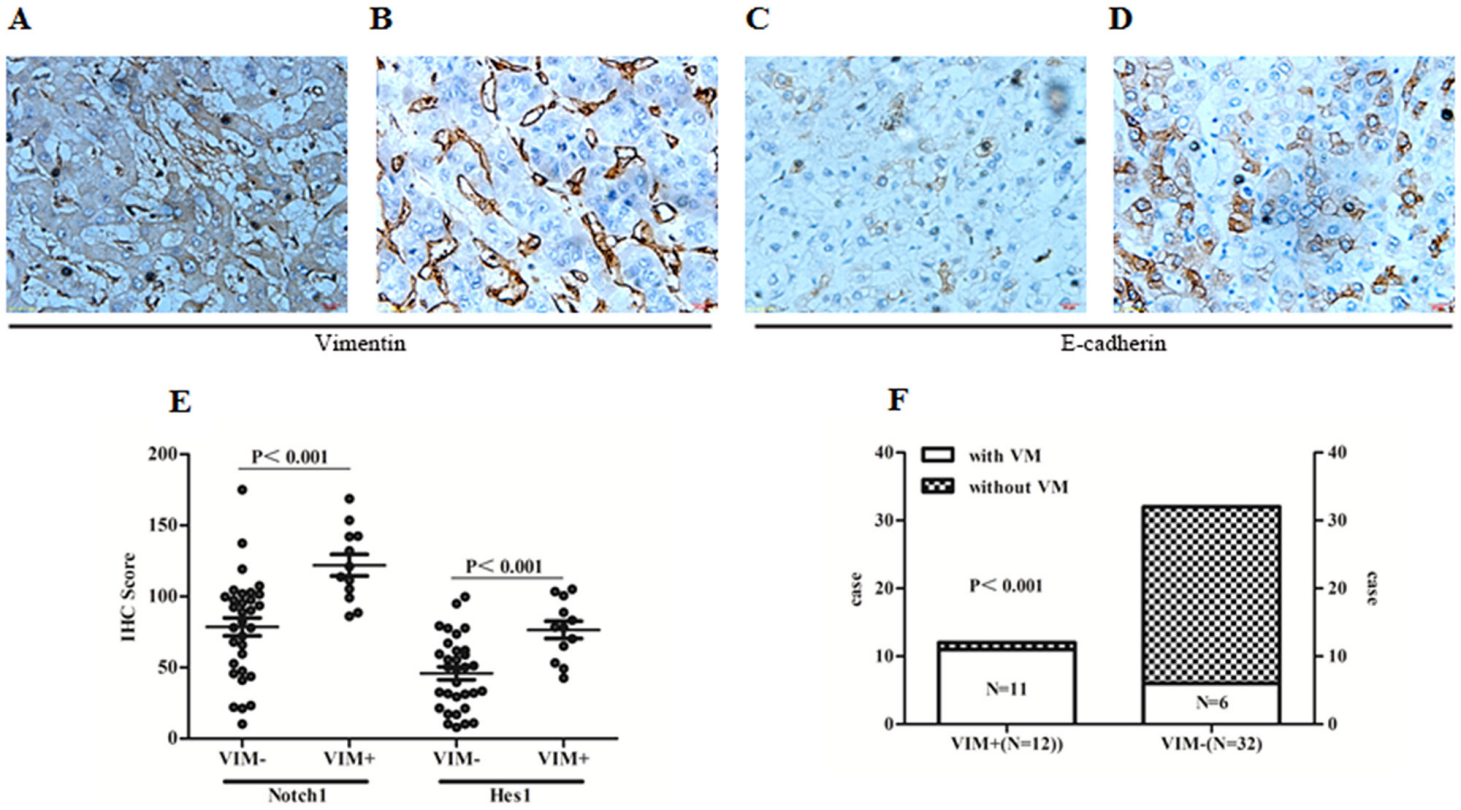
Notch1 associated with EMT related biomarkers in HCC tissues **A**. Positive Vimentin staining in tumor cells and microvascular wall. **B**. Positive vimentin staining only in microvascular wall. **C**. Expression of E-cadherin in HCC specimens with positive Vimentin staining in tumor cells. **D**. Expression of E-cadherin staining in HCC specimens with only positive Vimentin staining in microvascular wall. Original magnification 400×. **E**. Expression of Notch1 and Hes1 were elevated in those specimens with positive Vimentin staining in tumor cells (Notch1: 121.99±26.13 vs. 78.63±35.88; Hes1: 76.49±21.18 vs. 45.93±25.48). **F**. Distribution of cases with positive Vimentin staining in specimens with and without VM. VIM+: Positive Vimentin staining in tumor cells; VIM-: Negative Vimentin staining in tumor cells.

To further confirm the correlation between Notch1 and EMT signatures in patient's samples, Vimentin and E-Cadherin were examined with IHC. There were 12 cases in this study detected with positive vimentin staining not only in liver blood sinus but also in carcinoma cells (Figure 7A and 7B). Correspondingly, E-cadherin was found decreased in those specimens (Figure 7C). In the 12 Vimentin positive staining cases, the expression of Notch1 and Hes1 were found higher than those in another 32 cases with negative Vimentin staining (Figure 7E). In addition, 11 out of 17 specimens with VM structures were positively stained with Vimentin, while only 1 out of 27 cases without VM show positive vimentin staining (Figure 7F). These findings further suggested that EMT might be associated with VM formation, while Notch1 potentially contributed to the process.

### Manipulation on Notch1 expression influenced HCC xenograft growth and VM formation

**Figure 8:**
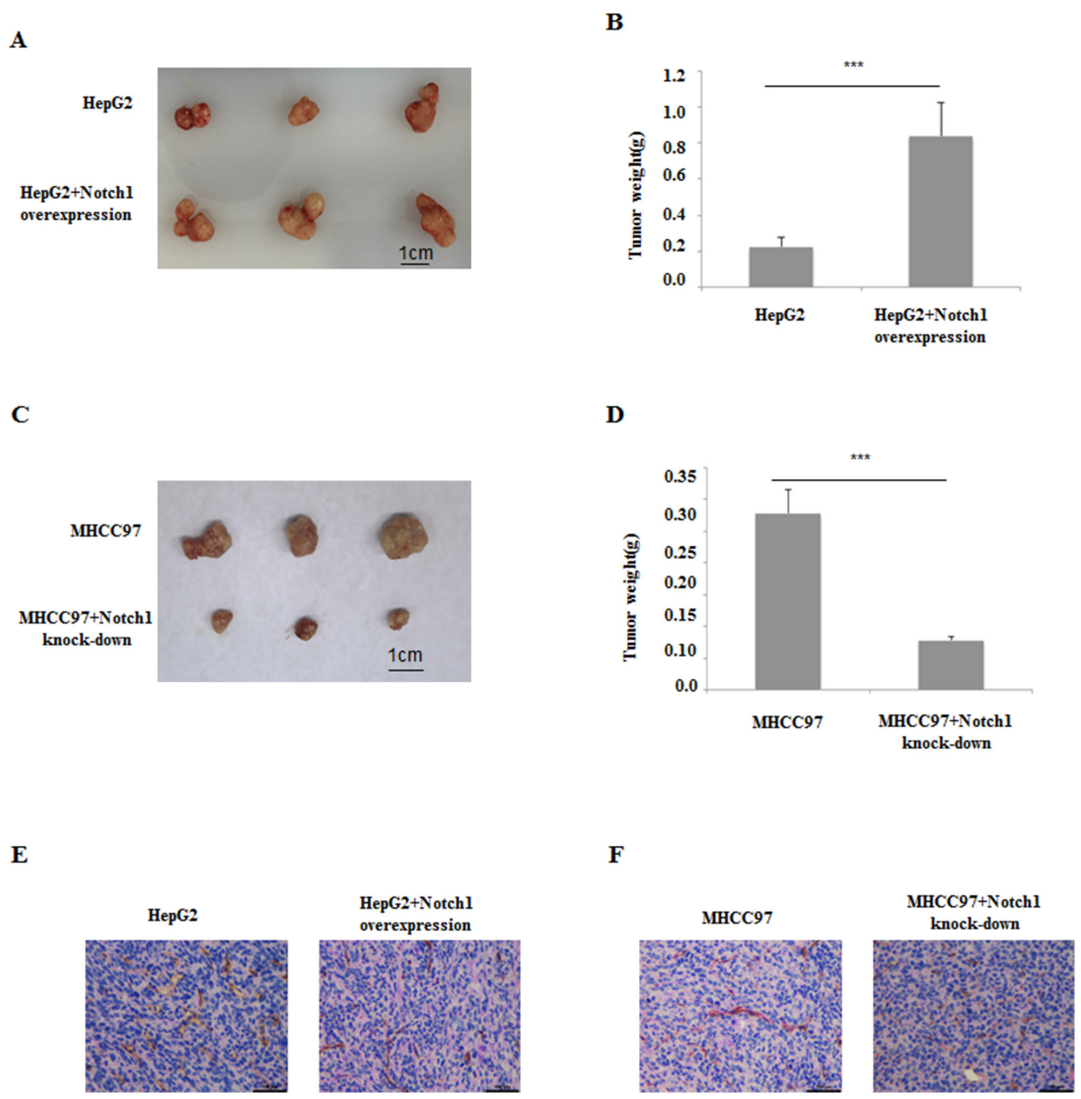
HCC xenografts with HepG2 cell and its Notch1 overexpression counterpart, MHCC97-H cell and its Notch1 knockdown counterpart **A** and **B**. Notch1 overexpression induced HepG2 to form bigger tumor *in vivo*. **C** and **D**. Notch1 knockdown induced MHCC97-H to form smaller tumor *in vivo*. **E**. CD34-PAS dual staining showed few VM in HepG2 xenografts compared with its Notch1 overexpression counterpart; **F**. CD34-PAS dual staining showed more VM in MHCC97-H xenografts compared with its Notch1 knockdown counterpart. Original magnification 400×.

Since the important roles of Notch1 in VM formation have been demonstrated by clinical analysis and *in vitro* experiments, its biological effect on HCC growth and VM formation was further examined using nude mice harboring HCC xenograft. HepG2 and it's Notch1 overexpression counterpart (HepG2/Notch1+), MHCC97-H and it's Notch1 knock-down counterpart (MHCC97-H/Notch1-) were subcutaneously injected into the athymic BALB/c mice. The tumors formed by HepG2/Notch1+ were larger and heavier than those formed by HepG2 (Figure 8A and 8B). While the tumors formed by MHCC97-H/Notch1- were obviously smaller and lighter than those formed by MHCC97-H (Figure 8C and 8D). CD34-PAS double staining indicated that more VM structures were found in HepG2/Notch1+ and MHCC97-H groups than those in HepG2 and MHCC97-H/Notch1- groups respectively (Figure 8E and 8F). These results indicated that Notch1 played an important role in HCC growth and VM formation, manipulation on Notch1 expression could disturb the disease progression.

## DISCUSSION

Hepatocellular carcinoma is one of the most common malignant tumors and the third-most common cause of cancer-related death world wide, especially in China [13]. Hypervascularity is one of the main characteristics of this disease and predicts poor prognosis [13]. However, the mechanisms of angiogenesis remain controversial [14]. Angiogenesis, whether physiological or pathological, is ‘switched on’ if the balance between proangiogenic factors and angiogenesis inhibitors tilts towards proangiogenic factors [15]. Sorafenib, the inhibitor of angiogenesis was demonstrated to improve survival in HCC patients [16, 17]. Although the outcome of Sorafenib treatment was not satisfying, it implys that angiogenesis was a meaningful target in drug development for HCC.

To better understand the mechanism of angiogenesis in HCC, we focus on the VM formation in HCC in the present study. We employ two HCC cell lines, HepG2 and MHCC97-H, with low and high Notch1 expression, respectively, to investigate the role of Notch1 in angiogenesis and HCC progression. We found that MHCC97-H cells, but not HepG2 cells form VM when they grow in matrigel *in vitro*. Interestingly, HepG2 cells gain the VM forming ability when they were overexpressed with Notch1, while knockdown Notch1 expression in MHCC97-H leads to the loss of VM forming ability of the cells. Similar results were found in the *in vivo* study. High expression of Notch1 in HepG2 cells promoted the xenograft growth in nude mice, with more VM formation in the tumor speciemens. The Notch signaling pathway regulates embryonic cell determination and differentiation as well as postnatal development [18, 19]. High Notch1 level has been linked to poor prognosis in breast cancer [20], where Notch1 has been shown to induce EMT [21, 22]. In this study, we observed that Notch1 was associated with the malignant behavior of hepatocellular carcinoma by analyzing the protein expression by IHC in the carcinoma specimens. To further understand wheather Notch1 is associated with EMT in regulating VM in HCC, we examined EMT markers in HCC cell lines, both MHCC97-H and HepG2. We found that Notch1 overexpression induced expression of mesenchymal biomarker Vimentin and suppressed expression of epithelial biomarker E-cadherin. Notch1 induced EMT phenomenon is mediated mainly by TGF-β1. MHCC97-H with high expression of Notch1 grows with VM formation when cultured in matrigel. In contrast, HepG2 with low expression of Notch1 was not able to do so. Both *in vivo* and *in vitro* results suggested that Notch1 expression was related to VM formation and EMT biomarkers expression, indicating that Notch1 promoted HCC progression through inducing VM formation by activating EMT pathway. Increasing evidences indicate that EMT is essential in the formation of VM [12, 23, 24], which is consistent with our result.

In summary, we demonstrated that Notch1 promoted HCC progression through VM formation induced by EMT signaling. Our results will provide a rational for targeting Notch1 for HCC new drug development.

## MATERIALS AND METHODS

### Tumor samples

HCC specimens and corresponding adjacent non-tumor specimens were obtained from patients who underwent resectable therapy at the Second People's Hospital of Taizhou (Taizhou, Jiangsu, China). None of these patients received antitumor treatments before the operation, and the diagnosis of HCC was pathologically confirmed. All samples were snap frozen in liquid nitrogen immediately after resection and stored at -80°C until processing. This study was approved by the institutional ethic committee of the Second People's Hospital of Taizhou (Taizhou, Jiangsu, China). All patients were requested to sign the informed consent.

### Immunohistochemistry (IHC)

All speciemens were fixed in neutral buffered formalin and embedded in paraffin. Slides were cut at 5μm, deparaffinized in xylene, and rehydrated in graded ethanol. Endogenous peroxidase activity was blocked with 3% hydrogen peroxide in 100% methanol for 10 minutes at room temperature. Sections were washed with phosphate-bufferedsaline (PBS), and then pretreated with citrate buffer (0.01mol/L citric acid, pH 6.0) for 20 minutes at 95°C in a microwave oven. After nonspecific binding sites were blocked by exposing them to 10% normal goat serum in PBS for 20 minutes, sections were incubated overnight at 4°C using a series of antibodies (please refer to Table 1). Following this incubation, the sections were rinsed with PBS and incubated with biotinylated goat anti-mouse IgG for 20 minutes at 37°C. The slides were then incubated with 3,3-diaminobenzidine chromogen for 5 to 10 minutes at room temperature and washed with distilled water. Finally, the sections were slightly counterstained with hematoxylin for 1 minute followed by dehydration and coverslip mounting. PBS was used in place of primary antibodies as negative controls.

**Table 1:** Information of primary antibodies used in immunohistochemical staining

Primary antibodies	Serial Number	Dilution	Source	Company
Notch1	4380	1:100	Rabbit	CST, Danforth, USA
Hes1	11988	1:5000	Rabbit	CST, Danforth, USA
Vimentin	5741	1:100	Rabbit	CST, Danforth, USA
E-cadherin	3195	1:400	Rabbit	CST, Danforth, USA
CD34	3569	1:50	Mouse	CST, Danforth, USA

CST, cell signaling technology.

### IHC Evaluation

All the slides were assessed by two independent pathologists who were blinded with the study background and outcomes. To quantify the IHC staining of Notch1, Hes1 and E-cadherin, the slides were imaged digitally with the same light exposure and evaluated by Image Pro Plus (IPP), a digitalized IHC scoring program (Media Cybernetics, San Diego, CA). For vimentin IHC staining, because the target staining location was in cytoplasm or cytomembrane other than microvascular wall, the staining located in HCC cells agreed by two pathologists was considered positive staining, conversely, it was considered negative staining if the staining was in microvascular wall.

### Cell culture

The human hepatocellular carcinoma cell line MHCC97-H and HepG2 were purchased from Fudan University (Shanghai, China). Huaman umbilic vein endogenous cell (HUVEC) line was purchased from Zhong Qiao Xin Zhou Biotech (SN: ZQ0113, Shanghai, China). All the cells were maintained in Dulbecco's modified Eagle medium (DMEM, GIBCO, Grand Island, NY, USA). Media contained 10% fetal bovine serum (Hyclone, Logan, USA), 100U/ml penicillin, 100mg/ml streptomycin, and 2mmol/l L-glutamine. Cells were cultured at 37°C in a condition with humidified atmosphere of 5% CO_2_.

### Cell invasion assay

The invasive ability of HCC cells were measured using 24-well transwell units with polycarbonate filters (pore size, 8μm) coated on the upper side with Matrigel (Becton Dickinson Labware, Bedford, MA, USA) according to the manufacturer's protocol. Briefly, 1 × 10^3^ cells in 100mL medium were seeded on the top chamber. The bottom chamber contained 10% fetal calf serum medium. After incubation for 24 hours, non-invasive cells were removed with a cotton swab. Cells that migrated to the bottom surface of the membrane were fixed in formaldehyde for 10 minutes, stained with 0.1% crystal violet solution, and counted under a microscope.

### Western blot analysis

Twenty micrograms of cell lysates were separated on 12% sodium dodecyl sulfate-polyacrylamide gel electrophoresis (SDS-PAGE) gels and then transferred onto nitrocellulose membranes. Specific monoclonal anti-Notch1 (CST, SN: 4380, delution: 1:2000), monoclonal anti-Hes1 (CST, SN: 11988, delution: 1:2000), monoclonal anti-Vimentin (CST, SN: 5741, delution: 1:2000), monoclonal anti-E-cadherin (CST, SN: 3195, delution: 1:2000) and monoclonal anti-β-actin (CST, SN: 8457, delution: 1:4000) were used. HRP conjugate immunoglobulin was used as a secondary antibody (Jackson ImmunoResearch Laboratories, West Grove, PA, USA). West Pico chemiluminescent (Pierce) was used as the substrate to visualize protein bands, which were quantified using densitometric image analysis software (Image Master VDS; Pharmacia Biotech). Normalization was made against β-actin expression.

### Reverse transcription polymerase chain reaction (RT-PCR)

Total RNA was isolated using Trizol reagent (Invitrogen, San Diego, CA, USA). First strand of cDNA was synthetized with reverse transcription kit (PrimeScript™ Synthesis kit, Takara Bio, Inc., Dalian, China). RT-PCR was performed using the SYBR Premix Ex Taq Kit (Takara Bio, Inc., Dalian, China ) on an Applied Biosystems 7500 Real Time PCR system (Applied Biosystems, White Plains, NY, USA). The β-actin was used as internal controlls. The experiment was performed in triplicate. Primers for Notch1, Hes1 and β-actin were described in Table 2. Data were shown as the fold changes.

**Table 2:** Primers and annealing temperatures for RT-PCR

Gene	Forward sequence	Reverse sequence	Annealing temperature
Notch1	5′-CACCCATGACCACTACCCAGTT-3′	5′-CCTCGGACCAATCAGAGATGTT-3′	65
Hes1	5’-TGATTTTGGATGCTCTGAAGAAAGATA-3’	5’-GCTGCAGGTTCCGGAGGT-3’	62
β-actin	5’-GAGGCACTCTTCCAGCCTTC-3’	5’-GGATGTCCACGTCACACTTC-3’	60

### Three-dimensional cultures

The following experiments were performed when cells reach 70–80% confluence in cultures. A 24-well tissue culture plate was used and every well was evenly coated with 200ul growth factor-reduced matrigel (BD Biosciences, Bedford, MA, USA), which was allowed to solidify at 37°C for 60 minutes, before cells were plated. The cell suspension was added (1 x10^5^ cells/well) on to the surface of the matrigel and incubated at 37°C for 48 hours. Cells were photographed using an Olympus IX51 inverted microscope (Olympus, Tokyo, Japan).

### In vivo tumor xenograft

Five-week-old male athymic BALB/c mice were used in the study, and maintained in a laminar flow cabinet under specific pathogen-free conditions. The animal experiments were approved by the Ethic Committee of Yang Zhou University (Yang Zhou, JiangSu, China). Cares were taken to minimize pains of the animals. HepG2 and its Notch1-overexpression counterpart, MHCC97-H and its Notch1-knockdown counterpart were harvested from subconfluent monolayer cultures by treatment with 0.25% trypsin, and washed with PBS twice before being resuspended in 100mL PBS. 1 × 10^6^ cells were subcutaneously injected into left armpit of nude mice. Four weeks after molding, animals were sacrificed and tumor weight was measured. tissues were fixed in formalin for further experiments.

### Lentivirus mediated Notch1 overexpression and lowexpression

Lentiviral vector carrying green fluorescent protein (GFP) for Notch1 overexpression and lowexpression were commercially constructed by Genechem Co., Ltd., (Shanghai, China). A total of 1 × 10^4^ HepG2 or MHCC97-H cells were suspended in 0.4ml complete DMEM in a 1.5ml tube, then 0.1ml vector stock (2×10^7^ IU/ml) was added and incubated at 37°C for 12 hours. Infected cells were then transferred into a 25cm^2^ tissue culture flask with 4ml of fresh complete medium and incubated continuously. Transduction efficiencies were evaluated at 72-hour after infection by counting GFP positive cells under a fluorescence microscope (Nikon Eclipse TE2000-U) and further confirmed by Western Blot.

### PAS-CD34 dual staining

IHC staining was applied to perform CD34 staining. The procedure was the same as above description in IHC part. PAS staining was peformed using PAS staining kit (SN: DG0005, Leagene Biotechnology Co., Ltd, Beijing, China). Brifly, after DAB reaction, sections were treated with 0.5% periodic acid solution for 10 minutes and rinsed with distilled water for 5 minutes, followed by staining in Schiff solution for 15-30 minutes. After rinsing with distilled water, sections were counterstained with hematoxylin, dehydrated, cleared and mounted.

### Statistical analysis

Statistical analyses were performed using SPSS 13.0 for Microsoft Windows (SPSS Inc., Chicago, IL, USA). Continuous variables were expressed as the means ± SD and were compared between groups by using the Student's t-test. Categorical variables were compared by using the Chi-Square test. The Mann–Whitney test was for non-normal distributive data. The analyses of time to postoperative recurrence was calculated by the Kaplan–Meier method, the differences in survival between groups were compared using the log-rank test. P<0.05 was considered statistically significant.
